# Controlling the Impact of *Helicobacter pylori*-Related Hyperhomocysteinemia on Neurodegeneration

**DOI:** 10.3390/medicina59030504

**Published:** 2023-03-04

**Authors:** Jannis Kountouras, Michael Doulberis, Apostolis Papaefthymiou, Stergios A. Polyzos, Christos Zavos, Evangelos Kazakos, Stergios Arapoglou, Foteini Kyrailidi, Maria C. Mouratidou, Marina Boziki, Elisabeth Vardaka

**Affiliations:** 1 Second Medical Clinic, School of Medicine, Ippokration Hospital, Aristotle University of Thessaloniki, 54642 Thessaloniki, Macedonia, Greece; 2 Division of Gastroenterology and Hepatology, Medical University Department, Kantonsspital Aarau, 5001 Aarau, Switzerland; 3 Pancreaticobiliary Medicine Unit, University College London Hospitals (UCLH), London W1W 6DN, UK; 4 First Laboratory of Pharmacology, School of Medicine, Aristotle University of Thessaloniki, 54124 Thessaloniki, Macedonia, Greece; 5 School of Healthcare Sciences, Midwifery Department, University of West Macedonia, Koila, 50100 Kozani, Macedonia, Greece; 6 Fifth Surgical Department, Medical School, Ippokration Hospital, Aristotle University of Thessaloniki, 54642 Thessaloniki, Macedonia, Greece; 7 2nd Neurology Department, School of Medicine, Faculty of Health Sciences, Aristotle University of Thessaloniki, AHEPA Hospital, 54124 Thessaloniki, Macedonia, Greece; 8 Department of Nutritional Sciences and Dietetics, School of Health Sciences, International Hellenic University, Alexander Campus, 57400 Thessaloniki, Macedonia, Greece

**Keywords:** *H. pylori*, hyperhomocysteinemia, brain cortical thinning, neurodegeneration, Alzheimer’s disease

## Abstract

*Helicobacter pylori* infection consists a high global burden affecting more than 50% of the world’s population. It is implicated, beyond substantiated local gastric pathologies, i.e., peptic ulcers and gastric cancer, in the pathophysiology of several neurodegenerative disorders, mainly by inducing hyperhomocysteinemia-related brain cortical thinning (BCT). BCT has been advocated as a possible biomarker associated with neurodegenerative central nervous system disorders such as Alzheimer’s disease, Parkinson’s disease, multiple sclerosis, and/or glaucoma, termed as “ocular Alzheimer’s disease”. According to the infection hypothesis in relation to neurodegeneration, *Helicobacter pylori* as non-commensal gut microbiome has been advocated as trigger and/or mediator of neurodegenerative diseases, such as the development of Alzheimer’s disease. Among others, *Helicobacter pylori*-related inflammatory mediators, defensins, autophagy, vitamin D, dietary factors, role of probiotics, and some pathogenetic considerations including relevant involved genes are discussed within this opinion article. In conclusion, by controlling the impact of *Helicobacter pylori*-related hyperhomocysteinemia on neurodegenerative disorders might offer benefits, and additional research is warranted to clarify this crucial topic currently representing a major worldwide burden.

## 1. Introduction

Recent data indicate the implications of microorganisms in the pathophysiology of neurodegeneration [1,2]. For instance, according to the Alzheimer’s disease (AD) pathogen theory, microorganisms operate as triggers, in conjunction with genetic variables, in starting the accumulation and/or processing of amyloid-β (Aβ), hyperphosphorylated tau proteins, and inflammation in the brains of AD [3]. Furthermore, brain cortical thinning (BCT), a hallmark feature mainly of brain aging, is linked with cognitive decline [4] and memory function disturbances [5]. BCT has been identified in many neurodegenerative disorders including AD [6], Parkinson’s disease (PD) [7], multiple sclerosis [8], or schizophrenia [9]. It is used as a biomarker of cognitive performance [10] to recognize mild cognitive-impairment subtypes [11]; to follow the clinical progression of disorders such as PD [7] or the progression of mild cognitive impairment to AD [10]; and to evaluate the effectiveness of interventional and rehabilitation procedures targeting improvements in sensory, motor, and cognitive functions [11].

Specifically, BCT, among other radiological MRI measurements, has been advocated as a possible biomarker associated with neurodegeneration either for primary neurodegenerative central nervous system (CNS) disorders such as AD [12] and/or PD [13] or other CNS disorders with a neurodegenerative pathological component, such as multiple sclerosis [13] and/or cardio-cerebrovascular-related dementia [14,15]. There are currently no diagnostic criteria for BCT; rather, longitudinal studies in the field provide estimates of normative rates in CNS pathology compared to age-related cortical thinning [16,17]. T1-weighted three-dimensional structural MRI scans are frequently used for this purpose, although advanced neuroimaging methodologies are continuously evolving [16]. 

According to the infection hypothesis in relation to neurodegeneration, microorganisms have been advocated as triggers and/or mediators of neurodegenerative diseases such as the development of AD [18]. The infection hypothesis of AD proposes that chronic infection with viral, bacterial, and/or fungal pathogens might be a trigger for AD onset during aging—probably via inflammatory processes—the risk of which seems predominantly high in apolipoprotein E (APOE) ε44 allele carriers. More specifically, proposed pathogens include latent neurotropic viruses (*Herpes simplex* virus type-1 and type-2; *Human herpes*- virus 4, 5, 6, and 7), gastrointestinal bacteria (*Helicobacter pylori*), periodontal bacteria (e.g., *Porphyromonas gingivalis*), pulmonary bacteria (e.g., *Chlamydophila pneumoniae*), spirochetes (e.g., *Borrelia burgdorferi*), and other infectious pathogens [18,19,20,21]. Over the last years, many detailed reviews have summarized the evidence about *herpes simplex virus type*-1, *Chlamydophila pneumoniae*, *Helicobacter pylori*, *Porphyromonas gingivalis,* and other oral infectious agents with the pathophysiology of AD [19]. 

In this regard, *Helicobacter pylori* (*H. pylori*) infection consists one of the most common chronic infections since it affects more than 50% of the world population [22]. Emerging evidence supports its involvement in the pathophysiology of many neurodegenerative disorders [21,23,24,25,26,27,28]. Specifically, found in gastric mucosa of ≥50% of humans worldwide, *H. pylori* can infect children, becoming chronic during adulthood if untreated; in most patients, *H. pylori* infections are acquired in childhood and, if untreated, persist throughout life [29], causing the chronic inflammatory process of the stomach lining and leading to many gastric as well as other upper and lower gastrointestinal and extra-gastrointestinal pathologies [30,31,32,33]. Therefore, epidemiological estimates of *H. pylori* prevalence refer to the untreated chronic *H. pylori* infection in adulthood. In this regard, chromic *H. pylori* infection is very common, with an assessed global mean prevalence of 58% (varies from 39.9% to 91.7%), partly due to immigrants coming from countries with a high prevalence of *H. pylori* infection [30,34,35]; it accounts of about 4.4 billion of the world’s *H. pylori* chronically infected individuals [36].

Despite the strong innate and adaptive immune responses, *H. pylori* exhibits long-term survival in the gastric mucosa. Its survival is intensely influenced by the ability of this bacterium to escape, disrupt, and manipulate the host immune responses. *H. pylori* employs several mechanisms to evade innate and adaptive immune systems’ immunity, allowing the bacterium to establish chronic infection [37]. *H. pylori*, by perturbing the gastric mucosa immune equilibrium, induces a microenvironment that favors gastric oncogenesis and evasion of immune surveillance [38]. This bacterium can escape from being recognized by innate immune receptors through changing its surface molecules. *H. pylori* can also block other innate recognition receptors by constraining downstream signaling pathways. Specifically, to the evasion of innate immune system mechanisms belong, among others, the following: neutralization of reactive oxygen and nitrogen species released by macrophages, alteration of cytokine secretion and the maturation of dendritic cells, and more passive systems such as the variability and the uniquely low immunogenicity of *H. pylori* lipopolysaccharide (LPS) [39]. Moreover, the expression of Lewis antigens in the LPS of *H. pylori* provides the bacterium with the ability to mimic host antigens and thus conceal itself from the immune system. *H. pylori* is also able to escape from the host adaptive immunity by modifying the function of the T lymphocytes. In this respect, *H. pylori* evasion from destruction by both the innate and adaptive immune responses may contribute to AD pathology [40].

It is important to note that diverse issues involved in the failure to eradicate *H. pylori* include, among others, *H. pylori* genotypes with high pathogenicity, the occurrence of point mutations, increased *H. pylori* burden, immunosuppressant characteristics, acidic gastric pH, intracellular occurrence of *H. pylori*, and/or biofilm creation linked with efflux pumps (transporters removing antibiotics from the cellular inner) [41]. *H. pylori* exhibits the ability to form biofilm in the stomach, which significantly decreases sensitivity to antibiotics and other antimicrobial agents and represents an independent mechanism that may be involved in the development and/or intensification of the antibiotic resistance. *H. pylori* biofilm appears to be a main reason of failure to eliminate infections triggered by *H. pylori*; antimicrobial agents are not capable of penetrating and destroying the biofilm; and *H. pylori* mutations resistant to antibiotics such as clarithromycin are commonly created in biofilms. Therefore, affecting the *H. pylori* biofilm-related pathologies can offer an efficient approach to avoid failures of *H. pylori* therapeutic regimens [41]. In this respect, exposure to key components of the innate immune system antimicrobial peptides, such as high human β-defensins (hβDs) 1–4 concentrations, significantly inhibits the capability for biofilm formation by *H. pylori,* a finding principally attributed to antibacterial action of hβDs [42]. Nevertheless, if *H. pylori* may access the brain, it could trigger the development and progression of neurodegenerative disorders, possibly by inducing defensin abnormal expression [43]. 

*H. pylori* also induces an intracellular niche that protects the bacterium from antibiotic eradication therapy, allowing persistence and recolonization. This intracellular niche may also contribute to bacterial evasion of the host immune responses [41]. In this regard, for instance, VDP, a direct vitamin D metabolite, possesses a *H. pylori*-specific antimicrobial ability through hβDs induction from infected macrophages, thus creating a promising therapeutic potential [41]. Notably, addition of vitamin D3 to the conventional clarithromycin-based triple therapeutic approach also offers a significant eradication rate of *H. pylori* infection. 

Apart from defensins, another key component of the innate immune system, namely human cathelicidin LL-37, is able to prevent *H. pylori* biofilm creation by inhibiting *H. pylori* adhesion, downregulating biofilm-related genes, suppressing quorum-sensing pathways, degrading the biofilm matrix, and suppressing the biofilm-residing cells [44]. 

Autophagy is involved in *H. pylori*-induced gastritis, and chronic *H. pylori* infection promotes gastric malignancy growth and progress by considerably weakening autophagy; although autophagy offers an anti-carcinogenic effect in normal tissues, the occurrence of an excess of altering triggers, such as *H. pylori* infection, prevents its activity, thereby promoting cancer development [41]. Interestingly, circulating monocytes (infected with *H. pylori* due to defective autophagy) through a disrupted blood–brain barrier (BBB) may trigger neurodegeneration [45]. The clarification of the interaction among *H. pylori* and autophagic process has driven investigators to examine the effect of the regulators of the autophagic process in order to control *H. pylori* infection in an effort to improve the rising antibiotic resistance. However, future research is warranted to elucidate in depth the above-mentioned considerations. 

Recurrence/reinfection of *H. pylori* infection can occur, and it can be categorized into two distinct mechanisms: recrudescence and reinfection [46]. The recrudescence is the reappearance of the original strain following initial eradication; it is considered to be a failure of eradication. The reinfection is infected with a new strain after initial eradication. The identification of the *H. pylori* strain needs the application of certain molecular fingerprinting techniques. These techniques are very complex procedures and demand high personnel and facilities resources. Therefore, currently, it cannot be widely introduced in clinical and scientific investigation. Some investigators consider that the recurrence of *H. pylori* infection less than 1 year after eradication is categorized as recrudescence but reinfection if it was more than 1 year [47,48]. Other investigators consider that the change of *H pylori* from negative to positive at 1 year following initial eradication might be caused by recurrence or reinfection, and reinfection is the principal cause of recurrence at 3 years after successful eradication [49]. A relative review and meta-analysis showed that the global annual recurrence, reinfection, and recrudescence rates of *H. pylori* were 4.3%, 3.1%, and 2.2%, respectively [50]. Risk factors of *H. pylori* reinfection include, among others, dental-plaque-related periodontitis, which is a multifactorial inflammatory disorder mediated by the host immune response to dental plaque [51,52]. Periodontitis is a risk factor for development of neurodegenerative disorders including AD, PD, or multiple sclerosis [53,54].

A large number of published studies cover *H. pylori*-preventing strategies, including dietary regimens. In general, dietary intervention to prevent *H. pylori*-related severe pathologies might be ideal because it carries no risk of bacterial resistance, safety, and rejuvenating action of atrophic gastritis. A few recent examples are the following: 

Soy product consumption significantly reduced the incidence of gastric cancer [55]. This could be attributed to the isoflavones (e.g., genistein, daidzein, and glycitein) that are abundant in soy products. In particular, genistein inhibits the growth of *H. pylori* and the activity of nuclear factor (NF)-κB, which is associated with gastrointestinal tract tumorigenesis [55].

Cranberry supplementation appears to improve the success of eradication of *H. pylori* infection, which represents the major cause of peptic ulcer disease and gastric cancer [56].

Vitamins A, C, D, E, B6, B9, and B12 as well as key minerals such as zinc, iron, copper, nickel, and selenium may be involved in *H. pylori* infection and eradication. The antioxidant activity of vitamins can reduce oxidative stress and further suppress *H. pylori*-induced inflammation. Moreover, in vitro studies demonstrated promising anti-*H. pylori* mechanisms for vitamin administration in a dose-dependent manner. Antioxidants including certain vitamins may exhibit a preventative and therapeutic potential against AD, thereby offering insights into the association between oxidative stress and neurodegenerative disorders [57].

The administration of probiotics seems to reduce *H. pylori* adhesion to gastric epithelial cells and prevent *H. pylori* colonization, particularly in children, or reinfection with *H. pylori* in high-risk adult patients [58]. In this respect, recent data indicate that probiotic supplementation also exhibits a highly significant effect on cognitive function in patients with both cognitive impairment and AD [59].

Maintaining good hygiene is effective strategy in preventing *H. pylori* infection [60]. Likewise, maintaining good hygiene (oral) is an effective strategy in improving the clinical condition of patients with AD [61].

However, such *H. pylori*-preventive strategies warrant further evaluation.

Interestingly, *H. pylori* is also related to metabolic syndrome (MetS)-systemic pathologies such as cardio-cerebrovascular and neurodegenerative diseases, the end outcomes of MetS [22,62].

In this respect, by introducing gastric mucosa histology representing the practical diagnostic gold standard for active burden of *H. pylori* infection, this infection, as in the case of the patients with neurodegenerative disorders, is also connected with BCT even in cognitively normal people. This connection persists despite further adjustment for MetS risk factors, thereby indicating that the active burden of *H. pylori* infection may contribute to neurodegeneration in cognitively normal people [63]. In contrast, *H. pylori* eradication regimens may ameliorate and/or prevent both local and systemic chronic *H. pylori* infection-related disorders. For instance, in a topical setting, *H. pylori* eradication seems to reduce and/or prevent autoimmune gastritis as indicated by improvement of gastric atrophy on follow-up, mucosa-associated lymphoid tissue lymphoma, gastric adenocarcinoma, as well as metachronous gastric cancer and preneoplastic lesions including atrophic gastritis and intestinal metaplasia [64,65]. In a systemic setting, *H. pylori* eradication may exert a positive effect on AD manifestations at 5-year survival endpoints [63]. Likewise, eradication of this bacterium could constrain the progression of clinically isolated syndrome to the development of multiple sclerosis [25]. Moreover, *H. pylori* eradication results in significant clinical improvement in the symptoms and better PD patient outcomes [66,67,68]. It is important to note that abnormal BCT may contribute to cognitive decline in patients with PD [69,70]. Furthermore, regarding glaucoma (defined as “ocular” AD), we reported a 2-year beneficial effect of *H. pylori* eradication on glaucoma progression, suggesting a possible causal link between this bacterium and glaucoma [45,71]. BCT may also contribute to the severity and progression of glaucoma, thereby serving as a novel marker for assessing the severity of glaucoma [72,73].

**Figure 1:**
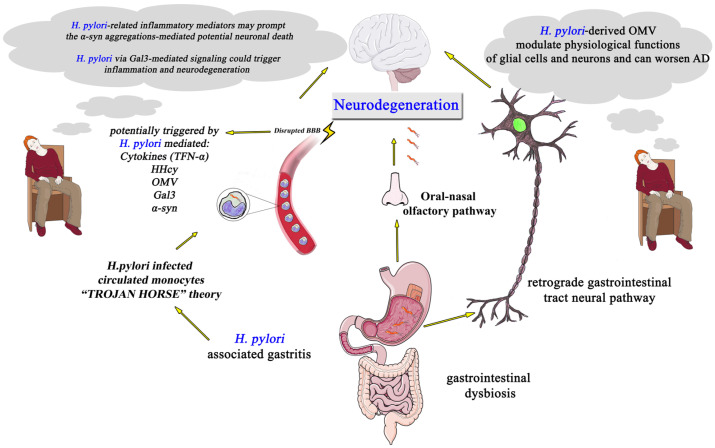
Schematic illustration of proposed pathophysiological pathways connecting *Helicobacter pylori* infection and neurodegeneration. AD, Alzheimer’s disease; α-syn, α-synuclein; BBB, blood–brain barrier; Gal-3, Galectin-3; *H. pylori, Helicobacter pylori*; HHcy, hyperhomocysteinemia; OMV, outer membrane vehicles; TNFα, tumor necrosis factor-α.

Various *H. pylori*-related potential mechanisms that trigger neurodegeneration include the following: *H. pylori*-induced systemic inflammatory responses; *H. pylori*-induced metabolic dysfunctions; *H. pylori*-related direct brain injury by inducing toxic materials or by attacking the brain via gastrointestinal retrograde axonal transport or oral-nasal olfactory pathways; circulated *H. pylori*-infected monocytes that enter the brain due to disruption of BBB (Trojan horse concept) and induce neurodegeneration; and/or *H. pylori*-related gastrointestinal microbiota dysbiosis that modifies the gut–brain axis toward neurodegeneration [63] (Figure 1). 

More specifically, *H. pylori* infection induces the release of a variety of molecules, such as IFN-γ, TNF-α, CRP, and IL-1, IL-6, and IL-10, all of which may promote apoptosis of neural tissue [74]. Additionally, structural components of *H. pylori* exhibit direct pro-inflammatory properties. For instance, *H. pylori* (2–20) peptides expressed by *H. pylori*, together with amyloid-β42, act as a ligand for formyl peptide receptors that play a key role in inflammation and lead to neurodegeneration. *H. pylori* (2–20)-mediated signaling results in increase of the vascular endothelial growth factor, a well-described mediator predisposing to vascular and other neurodegenerative dementias [75]. The direct pro-inflammatory properties of *H. pylori* infection components are also supported by experimental studies, according to which soluble surface fractions of *H. pylori* infection may lead to cognitive impairment by promoting the formation of Aβ42, thus compromising synaptic integrity and mediating neuronal damage [76]. Moreover, *H. pylori* infection associated with MetS may contribute to the vascular pathology, a well-described predisposing factor for neurodegenerative disease, by inducing increased plasma homocysteine levels and reduced serum 5-methyltetrahydrofolate and vitamin B-12, thus subsequently promoting endothelial damage. Interestingly, individuals with *H. pylori* infection exhibited cognitive impairment, the degree of which correlated with decreased 5-methyltetrahydrofolate levels [77]. *H. pylori* infection is also associated with increased tau-hyperphosphorylation, the key component of neurofibrillary tangles in AD [78]; it induces tau hyperphosphorylation, which is directly linked to the AD-associated neurodegeneration [79]. Intraperitoneal inoculation of *H. pylori* filtrate in rats provokes spatial learning and memory deficits, abnormal hippocampal dendritic spine maturation, and augmented presenilin-2 and Aβ42 in the brain’s hippocampus and cortex. Furthermore, *H. pylori* filtrate stimulates considerable tau hyperphosphorylation in mouse neuroblastoma N2a [76]. Since phosphorylation of tau is attenuated by the glycogen synthase kinase 3 inhibitor, this provides preliminary evidence for a beneficiary role of *H. pylori* eradication in AD and the need for verification by large-scale clinical trials [76]. Furthermore, we recently reviewed potential Galectin (Gal)-3-mediated mechanisms by which *H. pylori* may also promote neurodegeneration [27]. Gal-3 is a glycan-binding protein implicated in several physiologic and pathological processes including cell signaling, proliferation, and migration as well as the stimulation of immune response. *H. pylori* infection may promote neuroinflammation and neurodegeneration by several Gal-3-dependent pathways [27,80,81]. In addition, *H. pylori*-derived outer membrane vesicles modulate the physiological functions of glial cells and neurons and can worsen AD pathology [82]. *H. pylori* infection and/or gut microbiota alteration by promoting chronic inflammation, may influence both the occurrence and evolution of AD [83] (Figure 1). *H. pylori,* by releasing several inflammatory mediators (e.g., cytokines and chemokines induced by *H. pylori* infection), may induce BBB/blood–ocular barrier (BOB) breakdown, thereby being involved in the pathogenesis of neuropathies such as AD and glaucoma. TNF-α, induced by *H. pylori* infection, is involved in BBB disruption via a mechanism involving matrix metalloproteinase upregulation [62]. *H. pylori* VacA cytotoxin promotes intracellular survival of *H. pylori*, and activated monocytes (possibly infected with *H. pylori* due to defective autophagy) might access the brain (Trojan horse theory) via BBB/BOB disruption, thereby triggering the development and progression of neurodegenerative disorders, possibly by inducing additional defensin abnormal expression; when *H. pylori* accesses the brain, it may trigger defensin-related dendritic cells maturation and activation, leading to pro-inflammatory cytokine release by effector T cells, thereby promoting neuronal cell injury and death [24]. Furthermore, *H. pylori* could further contribute to neurodegenerative disorders’ pathogenesis by inducing MetS-related platelet–leukocyte aggregation, which is proposed to play pathophysiologic roles in AD; producing reactive oxygen species and circulating lipid peroxides implicated in AD pathobiology; and influencing the apoptotic process, a crucial cell death form in AD [66].

Therefore, controlling *H. pylori* infection may exhibit beneficial effects on neurodegeneration [22,62].

Additional critical triggers, particularly *H. pylori*-induced hyperhomocysteinemia, may play a crucial role in brain neurodegenerative disorders by provoking BCT in both normal males and females [84]. Since BCT, identified during the prodromal stages of AD, is a “candidate” biomarker implemented in AD clinical therapy trials, *H. pylori* eradication regimens, by affecting such BCT triggers, may exhibit a positive impact on cortical thickness, thereby signifying a relative protecting role in the development of brain-degenerative pathologies. 

Recently, genetic polymorphisms in genes that have been implicated in AD, a primary neurodegenerative disease and the most common cause of dementia worldwide [85], include, among others, *ABCA7* [86], *PICALM* [87], *CLU* [88], *SLC24A4/RIN3* [89], *ECHDC3* [90], *MTHFR* [91], as well as in insulin signaling such as *INSR* [92]. *MTHFR* mutations are associated with homocysteinemia, and increased homocysteine blood levels are considered to be an independent risk factor of cerebrovascular incidents. There are two common polymorphic genetic variants of the *MTHFR* gene that can lead to impaired functioning of the conversion of 5,10-methylenetetrahydrofolate to 5-methyltetrahydrofolate, a co-substrate for homocysteine remethylation to methionine, leading to hyperhomocysteinemia, which is a risk factor for dementia owing to an associated BBB dysfunction and subsequent small-vessel disease pathology [93]. Of note, the C677T polymorphism on the *MTHFR* gene can affect migraine susceptibility, with the resultant migraine being due to the increase in homocysteine levels in the blood [94]. Mutations in the gene SCIMP, a gene implicated in TLR-dependent activation of microglia, the innate immune system cell of the CNS, also have been recently identified in AD [95], thus shedding light onto possible mechanisms through which microbes and other infectious agents may affect the CNS in the context of neurodegeneration.

**Figure 2:**
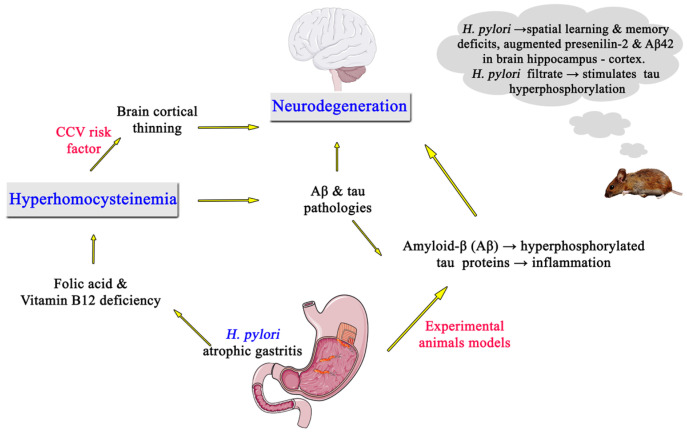
Schematic illustration of proposed pathophysiological mechanism (s) connecting *Helicobacter pylori* infection and neurodegeneration through the hyperhomocysteinemia pathway. CCV, cardio-cerebrovascular disorders; *H. pylori*, *Helicobacter pylori*.

Specifically, atrophic gastritis due to *H. pylori* infection-related MetS provokes deficiency of both vitamin B12 and folic acid, leading to hyperhomocysteinemia with consequent injury of the vascular endothelium [96]; *H. pylori*-provoked chronic gastritis leads to defective absorption of vitamin B12 and folate, resulting in methylation by 5-methyl-tetrahydrofolic acid malfunction and, therefore, in hyperhomocysteinemia [96] (Figure 2). More specifically, homocysteine, a sulfur-containing amino acid derived from methionine, is principally metabolized via methionine-synthase in the remethylation cycle, which is dependent on the presence of both vitamin B12 and folate as co-factors. The mechanisms of vitamin B12 malabsorption by *H. pylori* infection that result in hyperhomocysteinemia are unclear, but the following explanations are possible: First, as acid and pepsin are critical for splitting vitamin B12 from food binders and for its subsequent transfer to R binder in the stomach, the diminished acid secretion in *H. pylori*-induced gastritis may lead to a failure in absorption of food-bound vitamin B12. Second, *H. pylori*-induced gastritis could cause a secretory dysfunction of the intrinsic factor, thus leading to malabsorption of vitamin B12 and hyperhomocysteinemia. In addition, chronic *H. pylori* infection has been shown to decrease secretion of ascorbic acid from the gastric mucosa and to increase gastric pH. Low ascorbic acid in gastric juice or high pH of gastric juice have been demonstrated to produce reduced dietary folate absorption, also leading to hyperhomocysteinemia [97]. Of note, plasma homocysteine concentrations can range from 5 to 15 μmol/L in normal people [98], and elevated homocysteine levels of 15 μmol/L or higher are considered as indicators of hyperhomocysteinemia [99]. 

Hyperhomocysteinemia, as an atherogenic mediator, appears to be a risk factor for cardio-cerebrovascular diseases and AD, the end-points of MetS [62,100] (Figure 2). Moreover, the three “non-conventional” coronary artery disease risk factors, including hyperhomocysteinemia, hyperfibrinogenemia, and high lipoprotein-a, may stimulate the existence of arteriosclerosis and sequelae such as neurodegeneration [101,102]. More specifically, hyperhomocysteinemia has been involved in the pathophysiology of neurodegenerative diseases including AD. As an indicator of AD, increased homocysteine levels raise Aβ concentrations and/or deposition, thereby contributing to cognitive deterioration [79]. Increased homocysteine levels, with a subsequence insufficiency of methionine and S-Adenosyl-L-methionine and high S-Adenosyl-L-homocysteine, are linked with the reduced methylation capability. Inhibition of methylation reactions worsens the Aβ and tau pathologies also seen in *H. pylori*-associated AD [79,103]. Hyperfibrinogenemia also appears to be a risk factor of AD. An interaction between fibrinogen and Aβ in brain tissue with the possible creation of fibrinogen-Aβ plaques may be connected to cognitive dysfunction. High fibrinogen serum levels are associated with BCT [104], and fibrinogen plus Aβ complex formation is a hallmark of AD. Moreover, lipoprotein-a has been documented as a crucial factor in creation of Aβ plaques and exacerbation of AD.

Cognitively normal older individuals with elevated plasma concentrations of Aβ exhibit lower cognitive performance and higher homocysteine concentrations and BCT compared with those with reduced Aβ concentrations. Especially, such individuals with elevated Aβ1-40 exhibit a thinner temporal lobe, poorer self-perception of daily memory, and hyperhomocysteinemia [84]. Thinning of the temporal lobe appears to start eleven years before onset of cognitive impairment; BCT is a very sensitive indicator of neurodegenerative alterations, appearing decades before onset of AD clinical symptoms. Additional data also suggest that hyperhomocysteinemia is significantly associated with BCT, indicating cerebral atrophy in elderly asymptomatic populations [105] (Figure 2). Our findings show that active *H. pylori* infection appears to contribute to the pathogenesis of mild cognitive impairment, a prodromic condition predisposing for subsequent development of AD, by triggering the following sequence: chronic atrophic gastritis - hyperhomocysteinemia - mild cognitive impairment - AD development [23]. Since mild cognitive impairment is significantly connected with BCT [106], further research is necessary to evaluate whether lowering plasma homocysteine concentrations in both asymptomatic people and mild cognitive impairment patients with *H. pylori* infection may prevent neurodegenerative changes or delay the progression to the development of dementia.

Specifically, hyperhomocysteinemia appears to play an active role in the pathophysiology of mild cognitive impairment – AD sequence by triggering Aβ and tau pathologic conditions linked with synaptic dysfunction, neuroinflammatory process, and memory worsening (Figure 2). This finding signifies a potential novel strategy for the therapy of populations exhibiting this risk parameter [107]. In this respect, apart from increased levels of homocysteine, other *H. pylori*-associated MetS conditions provoke the mentioned mild cognitive impairment – AD sequence [62]. Experimental evidence indicates that only live *H. pylori* filtrates injection and not *Escherichia coli* induce in rats spatial learning and memory insufficiency [76]. Moreover, live *H. pylori* filtrates raise Aβ42 in the hippocampus and cortex by augmenting γ-secretase activity, thus triggering cognitive deterioration via interruption of the synaptic function [76] (Figure 2). Likewise, *H. pylori* filtrates stimulate tau hyperphosphorylation in rats at many AD-linked tau phosphorylation brain areas with activation of glycogen synthase kinase-3β (GSK-3β) [76] (Figure 2). 

In contrast, GSK-3 inhibitors seem to attenuate the *H. pylori*-provoked tau hyperphosphorylation. These data provide an indication for potential involvement of *H. pylori* infection in AD comparable tau pathology and suggest that *H. pylori* eradication might exert a beneficial effect against tauopathy [76]. The aforementioned experimental data seem to support our own clinical studies showing that *H. pylori* eradication may exert a positive effect on AD manifestations at 2- and 5-year survival endpoints [108]. Persistent *H. pylori* infection above 3 years results in hyperhomocysteinemia in healthy people [109]. Likewise, *H. pylori* eradication in a vitamin-B12-deficiency setting leads to increased vitamin B12 levels and reduced homocysteine blood levels [110]. Therefore, additional studies are needed to clarify the influence of eradication of this bacterium in the following sequence: hyperhomocysteinemia-linked asymptomatic setting - mild cognitive impairment - AD.

PD, a major worldwide health problem also associated with AD [111,112], is a neurodegenerative disease of the CNS affecting primarily dopaminergic neurons in the substantia nigra.

Alpha-synuclein (α-synuclein) is a protein participating in the transport of synaptic vesicles and the release of neurotransmitters in the synaptic cleft. Moreover, the α-synuclein protein is crucially involved in PD pathology, as the aggregation of α-synuclein is associated with neuron loss in PD. More specifically, α-synuclein misfolding leads to toxicity mediated by a-synuclein aggregates, namely protofibrils, that cause loss of synapses and neuronal death (Figure 1). In this respect, α-synuclein-associated mutations, for instance, in the SNCA gene, are related not only with familial PD but also predispose to sporadic disease. Pathological mechanisms that also contribute in PD evolution are mitochondrial dysfunction, the production of reactive oxygen species and other oxidative species, defective calcium homeostasis, endoplasmic reticulum/Golgi impairment, and microglia activation in the context of neuroinflammation [113]. Apart from the motor dysfunction in PD, non-motor symptoms have been the focus of extensive research, such as impairment of olfaction, mood disorders, and cognitive deficit [114,115]. Advanced neuroimaging biomarkers have been increasingly under study in the context of PD, and their association with cognitive function and other non-motor symptoms is under thorough investigation. More specifically, in PD, BCT has been associated with mild cognitive impairment [116] and rapid eye movement sleep behavior disorder [117], whereas it has been suggested as a possible biomarker with prognostic potential [118,119]. Changes in cortical thickness may lead to a dysfunction of resting-state functional connectivity, contributing to cognitive decline in patients with PD [69].

Recent meta-analysis indicates that both PD and AD are significantly linked with gastrointestinal disorders including *H. pylori* infection. Therefore, the adverse role of *H. pylori* in the pathophysiology of PD or AD should be evaluated to shed new light on the diagnosis and therapy of PD and AD [120]. 

Focusing on *H. pylori*-related PD, relative data indicate that *H. pylori* infection may play a role in PD pathophysiology. There is a strong association between *H. pylori* chronic infection-related gastric pathologies and exacerbation of PD symptoms [121]. A large cross-sectional study demonstrated a positive connection among *H. pylori* positivity (detected by ^13^C-urea breath test) and PD motor severity [121]. Likewise, *H. pylori* infection and MetS are important risk factors for PD [66]. Apoptotic microglia-associated nerve cell death seems to underlie PD and other common neurological conditions including AD, multiple sclerosis, and glaucoma, which are also associated with *H. pylori* infection [66].

In this regard, hyperhomocysteinemia could drive PD development and progression via many pathways involving apoptosis, oxidative stress, mitochondrial dysfunction, and DNA damage in nerve cells [122]. In PD, hyperhomocysteinemia seems to be connected with cognitive performance and structural damage in the cerebral cortex [123]. Hyperhomocysteinemia is a risk factor for cognitive decline in PD [124]. Thus, hyperhomocysteinemia during PD development and progression offers a novel strategy for the diagnosis and treatment of PD [122]. Relative data indicate the occurrence of hyperhomocysteinemia in levodopa-treated PD patients, whereas vitamin B12 and folate supplementation may decrease homocysteine levels [125]. Likewise, *H. pylori* eradication in patients with PD might improve the bioavailability of L-3,4-dihydroxyphenylalanine (L-dopa), a precursor of dopamine used as a treatment for PD, and reduce motor fluctuations [111]. In addition, *H. pylori,* which selectively infects and colonizes predominantly the gastric epithelium and oral cavity [126], is a risk factor for periodontitis [127]; periodontitis, beyond AD [53], is linked with PD [128,129]. In contrast, *H. pylori* eradication and treatment of periodontitis are beneficial for the prevention of PD and dementia [127].

Equivalent data may also be obtained for clinically isolated syndrome - multiple sclerosis sequence [25,28,130]. Indeed, our studies show that active *H. pylori* infection, apart from multiple sclerosis [28,130], is also common in clinically isolated syndrome, a prodromic condition predisposing for subsequent development of multiple sclerosis, accompanied by increased homocysteine levels. Moreover, eradication of this bacterium could constrain the progression of clinically isolated syndrome to the development of multiple sclerosis [25]; BCT is observed in both clinically isolated syndrome and multiple sclerosis [130]. In addition, recent data indicate that hyperhomocysteinemia, beyond cardiovascular disorders, appears to be an important factor involved in the pathogenesis of such an autoimmune disease of the CNS system as multiple sclerosis; it plays a role in endothelial dysfunction in multiple sclerosis [131]. Hyperhomocysteinemia is linked with high process activity and disease progression by involving in the mechanisms of neurodegeneration observed in multiple sclerosis. Thus, homocysteine concentration may be a potential marker predicting the course of the disease [132]. Another study reported higher homocysteine blood levels in patients with progressive multiple sclerosis compared to patients with relapsing-remitting multiple sclerosis [133]. Of note, progressive multiple sclerosis is a form of the disease typically considered to exhibit higher neurodegenerative components in terms of underlying pathology, whereas relapsing-remitting multiple sclerosis is primarily characterized by increased relative contribution of neuroinflammation in the disease pathology [134]. In this respect, the findings of the study underline a possible mechanism by which *H. pylori* infection and *H. pylori* infection-related hyperhomocysteinemia may contribute to neurodegeneration in the context of multiple sclerosis.

Glaucoma, the leading cause of irreversible blindness worldwide, is linked with both PD and AD [135]. 

Specifically, active *H. pylori* infection, beyond PD, is also associated with primary open-angle glaucoma, which has been called “ocular AD”, and pseudo-exfoliative glaucoma in some populations studied [136,137,138]. Moreover, *H. pylori* eradication seems to improve glaucoma parameters, thereby signifying a potential causal relationship between *H. pylori* infection and glaucoma [136]. Glaucoma induces the death of retinal ganglion cells and their axons and appears to be the second most common reason of blindness worldwide [138]. Although glaucoma is frequently considered an eye-only disorder, relative data suggest the involvement of the brain’s visual system in glaucoma. Post-mortem histology has disclosed that signs of neurodegeneration and associated metabolic changes are not restricted to retinal ganglion cells and the optic nerve but encompass upstream parts of the central visual pathway including the visual cortex. According to the mentioned BCT analyses, cortical degeneration within the visual areas becomes progressively aggravated with the augmenting severity of primary open-angle glaucoma [139]. The cortical thinning of visual areas might play a key role in the progression of glaucoma and can be a novel biomarker for assessing the severity of glaucoma [139]. Moreover, the inner retinal layer thinning, optic nerve cupping, and reduced visual cortex activity may occur before patients show visual field impairment [72].

In this respect, hyperhomocysteinemia may play a role in the pathogenesis of retinal diseases, especially glaucoma and age-related macular degeneration [140]. Oxidative stress and mitochondrial dysfunction are main mediators involved in the pathogenesis of neurodegenerative and in inner retinal neurodegenerative disorders. These mediators are also mediators of hyperhomocysteinemia-induced neuronal retinal ganglion cells death [141]; and oxidative stress is a major pathogenic mechanism related to hyperhomocysteinemia. Particularly, as in the case of *H. pylori* infection [137,138,142], hyperhomocysteinemia induces endothelial dysfunction and is associated with primary open-angle glaucoma and/or pseudo-exfoliation glaucoma, thereby signifying the potential prevention and therapy of glaucoma by regulating homocysteine levels [143]. Moreover, nutritional disturbances seem to be connected with an augmented risk of glaucoma [139], and the treatment of hyperhomocysteinemia by receiving low-cost vitamin B12 and folic acid regimens may prevent vascular abnormalities of this disorder [144]. Of note, early and long-term vitamin B12 supplementation may offer benefits against cognitive decline [145]. Nevertheless, whether *H. pylori* eradication, beyond other glaucoma parameters, offers an additional benefit regarding hyperhomocysteinemia-associated glaucoma remains to be elucidated in the future.

Finally, BCT in schizophrenia might be correlated with subsequent suicidal behavior [146]. In this regard, hyperhomocysteinemia appears to play a role in the pathophysiology of schizophrenia; hyperhomocysteinemia is correlated with negative symptoms of schizophrenia [147], and it may serve as a prognostic marker for schizophrenia [148]. Likewise, *H. pylori* has been proposed to play a role as a potential environmental factor for pathogenesis of schizophrenia [149], and *H. pylori* infection, associated with homocysteine changes, might contribute to schizophrenia development [150]. Moreover, both schizophrenia and *H. pylori* infection have been correlated with malnutrition [151,152], and *H. pylori* infection, among others, induces the reduced bioavailability or the malabsorption of essential nutrients such as the mentioned vitamin B12 and folate, leading to hyperhomocysteinemia [151]. Vitamin supplementation, especially with low-cost vitamin B12, folate, and vitamin D, could offer significant benefits in the treatment of schizophrenia [153] and could improve the *H. pylori* eradication rate [154]. Nevertheless, whether *H. pylori* eradication may also offer benefit in the treatment of schizophrenia requires further investigation.

In conclusion, controlling the impact of *H. pylori*-related hyperhomocysteinemia on such neurodegenerative disorders might offer benefits, and additional research is warranted to clarify this crucial topic currently representing a major global burden.

## Data Availability

Not applicable for opinion article.
